# Seed Morphology of *Allium* L. Endemic Species from Section *Schoenoprasum* (Amaryllidaceae) in Eastern Kazakhstan

**DOI:** 10.3390/biology14091230

**Published:** 2025-09-09

**Authors:** Aidar Sumbembayev, Olga Lagus, Alevtina Danilova, Zhanar Aimenova, Ainur Seilkhan, Zhanar Takiyeva, Agnieszka Rewicz, Sławomir Nowak

**Affiliations:** 1 Altai Botanical Garden, Ridder 071300, Kazakhstanaimenova.zhanara@gmail.com (Z.A.); 2 Department of Biodiversity and Bioresources, Al-Farabi Kazakh National University, Almaty 050040, Kazakhstan; 3 Laboratory of Phytochemistry, M. Auezov South Kazakhstan University, Tauke Khan Avenue 5, Shymkent 160012, Kazakhstan; 4 Faculty of Natural Sciences and Geography, Abai Kazakh National Pedagogical University, Almaty 050010, Kazakhstan; 5 Department of Geobotany and Plant Ecology, University of Lodz, 90-237 Lodz, Poland; 6 Department of Plant Taxonomy and Nature Conservation, Faculty of Biology, University of Gdansk, 80-308 Gdańsk, Poland; slawomir.nowak@ug.edu.pl

**Keywords:** taxonomy, conservation, *Allium ledebourianum*, *Allium ivasczenkoae*, *Allium schoenoprasum*, *Allium ubinicum*

## Abstract

The genus *Allium* is highly diverse and ecologically significant in Eastern Kazakhstan, with several rare and endemic species belonging to the section *Schoenoprasum*. This study provides a comparative analysis of the seed morphology of four species from this section: *A. ledebourianum*, *A. ivasczenkoae*, *A. schoenoprasum*, and *A. ubinicum*. Seeds were collected from their natural habitats and analyzed through morphometric measurements, surface structure assessments, and environmental correlation. Distinct differences in seed length, width, thickness, and weight were identified, with *A. ubinicum* displaying the most pronounced divergence in size and mass. Qualitative traits, including surface texture, micropyle visibility, and chalaza structure, were also recorded. Principal component and cluster analyses revealed clear separation between the species, notably distinguishing *A. ubinicum* from *A. schoenoprasum*, challenging their previously assumed synonymy. Environmental variables such as light, moisture, and soil chemistry showed significant correlations with seed dimensions, suggesting that environmental conditions contribute to morphological differentiation. Although the micromorphological features observed under scanning electron microscopy were generally similar, the macromorphological traits proved to be taxonomically informative. The larger and heavier seeds of *A. ubinicum* suggest regional divergence or adaptive evolution, reinforcing its classification as a separate taxon. These findings contribute to the refinement of the species delimitation process within *Schoenoprasum* and underscore the role of seed traits in both taxonomic research and conservation strategies in floristically rich but understudied regions like Eastern Kazakhstan.

## Simple Summary

The *Allium* genus, including onions and chives, is diverse and vital in Eastern Kazakhstan, especially in the *Schoenoprasum* section with rare species. This study examines seed shapes and sizes of four species—*A. ledebourianum*, *A. ivasczenkoae*, *A. schoenoprasum*, and *A. ubinicum*—to clarify their differences and challenge if *A. ubinicum* is distinct from *A. schoenoprasum*. By analyzing seed measurements, textures, and environmental impacts like light and soil, the researchers aim to improve plant identification and conservation. Findings of larger seeds of *A. ubinicum* suggesting unique adaptations could guide future classification and protect these plants in unexplored regions like Eastern Kazakhstan.

## 1. Introduction

The genus *Allium* L., with over 1000–1200 species recognized globally [1,2], is the largest genus in the Amaryllidaceae family and includes vital crop plants such as onion (*A. cepa* L.), garlic (*A. sativum* L.), leek (*A. ampeloprasum* L.), and chives (*A. schoenoprasum* L.) [3,4,5]. Beyond their economic role, *Allium* species are valued as medicinal and ornamental plants [6,7] and are well known for diverse biologically active compounds, including allicin, antioxidants, and organosulfur molecules with anti-inflammatory and antimicrobial activity [8].

Numerous studies have explored *Allium* in terms of genetics [2,9,10,11,12], distribution [13,14,15], phytochemistry [16,17,18], and morphology [19,20,21]. Among morphological features, seeds are particularly relevant because generative traits often hold taxonomic and phylogenetic value [22,23,24,25,26,27]. Despite this, while seed morphology has been extensively studied in territories adjacent to Kazakhstan [28,29,30], investigations within Kazakhstan remain limited [31,32,33,34]. Celep et al. (2012) [22] demonstrated that macro- and micro-morphological seed characters (e.g., testa ornamentation, cell shape, wall undulations) carry diagnostic and phylogenetic significance across multiple *Allium* sections, these comprehensive studies have not covered taxa endemic to Eastern Kazakhstan. Existing local research has largely centered on population status and genetic diversity of individual species such as *A. altaicum* [6,35] and *A. ledebourianum* [11], leaving a gap in seed-based systematics for the region.

Kazakhstan harbors over 120 *Allium* species [1,36], with 45 occurring in Eastern Kazakhstan across the subgenera *Rhizirideum* (Koch) Wendelbo, *Allium*, *Mollium* (Koch) Wendelbo, and *Melanocrommyum* (Webb. et Berth.) Rouy [37]. Many are narrow endemics, including *A. ledebourianum*, *A. azutavicum*, *A. ivasczenkoae*, *A. ubinicum*, *A. robustum*, *A. platyspathum*, and *A. zaissanicum* [35,37]. Within section *Schoenoprasum* Dumort., Eastern Kazakhstan is home to *A. ledebourianum*, *A. ivasczenkoae*, *A. ubinicum*, and *A. schoenoprasum*. Notably, *A. schoenoprasum* populations on the Kalbinsky Ridge represent a Pleistocene glacial relic [37].

Section *Schoenoprasum* is taxonomically and ecologically significant. Its species are adapted to diverse temperate habitats, from wetlands to rocky slopes [7], and serve as models for studying ecological adaptation. They have been central to phylogenetic and evolutionary analyses owing to their distinct genetic features [2,38]. Economically, *A. schoenoprasum* is cultivated as a food crop and medicinal herb [7], with applications in breeding programs for improved flavor, stress tolerance, and pest resistance [39]. Conservation concerns are also relevant since the section includes rare, endemic taxa central to biodiversity management [38].

Within *Allium*, the morphology of generative organs (flowers, fruits, seeds) remains a cornerstone of species delimitation [40,41,42]. Seed morphology, in particular, is increasingly recognized as an effective diagnostic tool for identifying rare and endemic taxa [43,44,45]. While previous studies have examined seed morphology across *Allium* species, they have rarely included taxa from Eastern Kazakhstan. The inclusion of *A. ubinicum*, *A. schoenoprasum*, and related taxa provides new insight into species boundaries within section *Schoenoprasum*, addressing unresolved taxonomic questions and expanding the geographic scope of comparative *Allium* seed morphology research.

A central unresolved issue concerns the taxonomic distinctness of *A. ubinicum* versus *A. schoenoprasum*. The taxonomic status of *A. ubinicum* has long been debated, with some treatments regarding it as a variant of *A. schoenoprasum* [2], while others recognize it as a distinct species based on morphological and environmental traits [46]. Field observations in Eastern Kazakhstan suggest morphological and ecological divergence that may warrant its recognition as a separate species [1,37]. This uncertainty has direct implications for taxonomy, biodiversity conservation, and evolutionary interpretation.

Therefore, this study aims to characterize the seed morphology and quantitative morphometrics of *Allium* species in the section *Schoenoprasum* from Eastern Kazakhstan. Specifically, we investigate whether seed traits can serve as reliable taxonomic markers to differentiate closely related taxa, with particular focus on clarifying the status of *A. ubinicum* relative to *A. schoenoprasum*. The inclusion of the four targeted *Schoenoprasum* species in our study fills this geographic and taxonomic gap, enriching the current understanding of seed morphology across Central Asian *Allium* diversity. By integrating high-resolution imaging with morphometric analysis, we address a broader question of how seed characters contribute to evolutionary understanding and conservation assessment of *Allium* in an ecologically dynamic region. Theoretically, the study advances knowledge on the role of seed morphology in species delimitation within complex genera and contributes to resolving taxonomic uncertainties in underexplored regions. Practically, the findings provide baseline morphological criteria useful for field identification, ex situ conservation, and the sustainable utilization of endemic *Allium* resources in Kazakhstan.

## 2. Materials and Methods

### 2.1. Collection Sites

**Figure 1:**
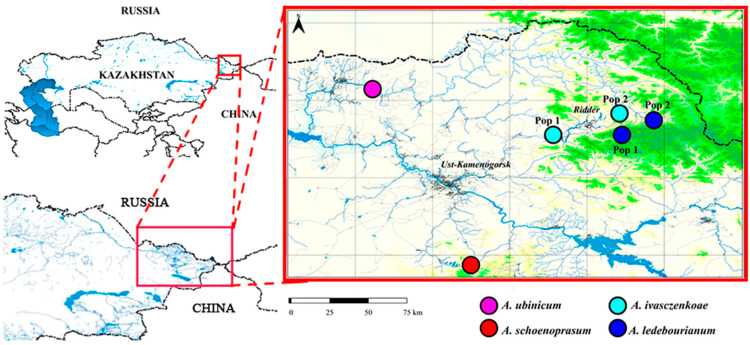
Geographic locations of seed collection from populations of *Allium* (section *Schoenoprasum*) in Eastern Kazakhstan. Colored dots indicate four species.

Seeds of four endemic species of the genus *Allium* from the section *Schoenoprasum* were collected in July 2023 in Eastern Kazakhstan (Figure 1). Studying the habitats revealed four species of the *Schoenoprasum* section, represented by 6 populations (Table 1). These 4 species belong to the section *Schoenoprasum* Dumort. of the subgenus *Rhizirideum* (Koch) Wendelbo, within the genus *Allium* L. of the family Amaryllidaceae J.St.-Hil.

### 2.2. Biometric Analysis of Seeds

**Figure 2:**
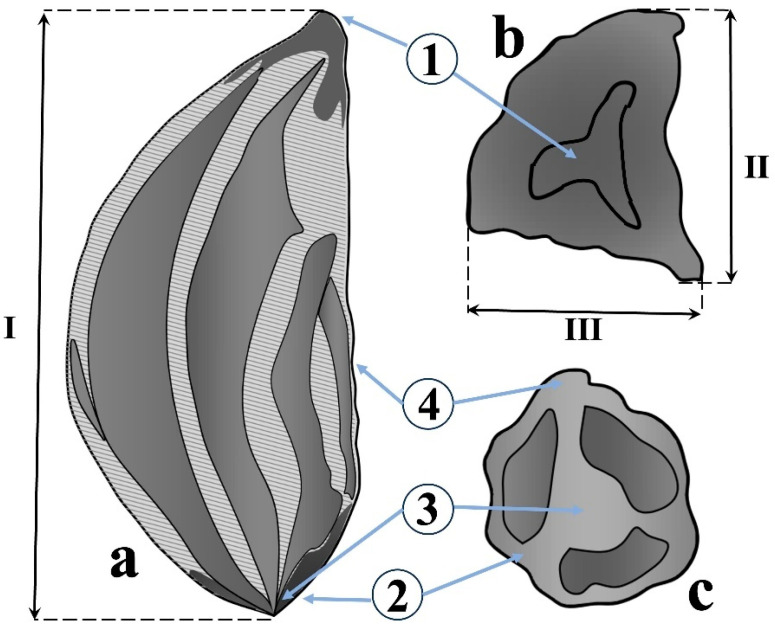
Schematic structure of seeds of *Allium* species. (**a**)—main view; (**b**)—top view; (**c**)—bottom view. Explanation: 1—micropyle, 2—chalazal end (chalaza), 3—seed hilum (hilum), 4—seed suture (raphe), I—length, II—width, III—thickness.

The freshly collected seeds were elastic, moist, and black, with a smooth, shiny surface. After several days of dry storage in paper bags at 16 °C, the seeds hardened, and their surface became rough and dull. Only fully formed and healthy seeds were used for analysis. For morphometric analyses, 30 seeds were randomly selected from each population, ensuring equal representation across all statistical models. During the external examination, common characteristics of the seeds for each species of the section *Schoenoprasum* were identified. The main quantitative parameters were length (mm), width (mm), thickness (mm), and the weight of 1000 seeds (g). The qualitative parameters of the seeds were also evaluated, including shape and color, surface relief, micropyle structure, chalaza, seed hilum, and suture (Figure 2).

The shape and surface of the seeds were described according to the method of Martin, 1946 [47]; the seed sizes (length, width, and thickness) were measured using a Magus A18T stereomicroscope (MAGUS, Moscow, Russia); and the seed color was determined using the MacAdam color scale (1974) [48]. Species nomenclature follows POWO [1] and Abdulin (1999) [36]. The systematics of the genus and sectional division are based on the works of R.V. Kamelin (1973) [49] and Friesen et al. (2006) [2].

The seeds were fixed in 2.5% glutaraldehyde (0.1 M phosphate buffer, pH 7.2) for 24 h at 4 °C, rinsed in buffer, and dehydrated through a graded ethanol series (30–100%). Critical-point drying was followed by brief rehydration in 100% ethanol to prevent surface collapse. The dried seeds were coated with a thin layer of gold using a sputter coater. Scanning electron microscopy (SEM) was conducted with the Phenom Pro X microscope (Thermo Fisher Scientific, Waltham, MA, USA). Three-dimensional models of the seed surface ultrastructure were created using the 3D Roughness Reconstruction function available for the Phenom Electron Microscope (Phenom-World, Eindhoven, The Netherlands). Schematic diagrams of seed structures were manually drawn in AutoCAD LT 2024 (Autodesk Inc., San Francisco, CA, USA) based on high-resolution images and morphometric measurements of the seeds obtained through stereomicroscopy.

The following environmental conditions of the studied populations’ growing areas were assessed visually according to the Ellenberg Indicator Values (EIVs) [50]: light (L), temperature (T), moisture (M), soil reaction (pH, R), soil nutrient richness (N), and salinity (S) (Table S1).

### 2.3. Statistics

Multivariate statistical analyses were performed to investigate interspecific variation and evaluate diagnostic morphological traits of the studied *Allium* species. PCA and correlation analyses were carried out using R software version 4.0.1 within the RStudio environment (version 1.3.1082), employing the stats, factoextra, and corrplot packages. A hierarchical clustering analysis was also performed using Ward’s method based on Euclidean distances to construct a sectional dendrogram. The dendrogram was created using the hclust function in R version 4.0.1 and visualized with the ggplot2 version 3.5.2 package.

## 3. Results

### 3.1. External Morphology and Seed Metrics

**Figure 3:**
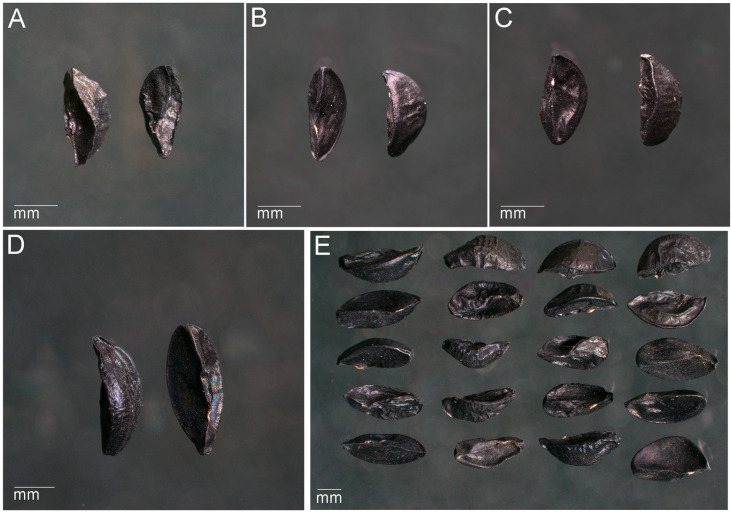
Seeds of 4 species of the section *Schoenoprasum*: (**A**)—*A. schoenoprasum*, (**B**)—*A. ivasczenkoae*, (**C**)—*A. ledebourianum*, (**D**)—*A. ubinicum*, (**E**)—comparison of seeds of the section *Schoenoprasum* from the Kazakh Altai (from left to right: *A. schoenoprasum*, *A. ivasczenkoae*, *A. ledebourianum*, and *A. ubinicum*).

Examining the external seed morphology revealed distinct diagnostic characteristics for each species that are taxonomically informative and contribute to differentiation and classification within this section (Figure 3).

Analyzing the quantitative morphological traits of the *Allium* seeds—including the length, width, and thickness—revealed generally low to moderate levels of variability among the studied taxa (Table 2).

*A. ubinicum* exhibited the longest seeds, ranging from 3.05 to 4.00 mm (mean 3.44 ± 0.14 mm, CV 10.43%), surpassing *A. ledebourianum* (2.25–3.50 mm, mean 3.04 ± 0.09 mm, CV 7.93%), *A. ivasczenkoae* (2.95–3.90 mm, mean 3.27 ± 0.08 mm, CV 6.50%), and *A. schoenoprasum* (3.03–3.40 mm, mean 3.06 ± 0.08 mm, CV 7.30%). Seed width was greatest in *A. ivasczenkoae* (1.25–1.90 mm, mean 1.47 ± 0.05 mm, CV 8.46%), followed by *A. ledebourianum* (1.34–1.80 mm, mean 1.54 ± 0.06 mm, CV 9.35%), *A. ubinicum* (1.17–1.85 mm, mean 1.49 ± 0.06 mm, CV 10.87%), and *A. schoenoprasum* (1.15–1.65 mm, mean 1.35 ± 0.05 mm, CV 9.70%). Seed thickness was highest in *A. ledebourianum* (1.51–1.74 mm, mean 1.71 ± 0.04 mm, CV 6.44%), compared to *A. ivasczenkoae* (1.35–1.75 mm, mean 1.54 ± 0.06 mm, CV 6.11%), *A. ubinicum* (1.27–1.70 mm, mean 1.51 ± 0.05 mm, CV 8.54%), and *A. schoenoprasum* (1.20–1.62 mm, mean 1.40 ± 0.04 mm, CV 7.00%). The heaviest 1000-seed weight was observed in *A. ubinicum* (1.35 g), followed by *A. ledebourianum* (1.12–1.20 g), *A. schoenoprasum* (0.88 g), and *A. ivasczenkoae* (0.60–0.96 g), indicating *A. ubinicum* as the taxon with the heaviest seeds.

Population comparisons revealed no significant differences within species. For *A. ledebourianum*, both populations showed similar ranges with overlapping means and coefficients of variation. Similarly, *A. ivasczenkoae* populations exhibited consistent ranges, as did *A. ubinicum* and *A. schoenoprasum*, with no notable inter-population variation in means or CV values. Overall, statistical parameters (MSE, CV%, P%) revealed considerable variability among species, with *A. ubinicum* showing the greatest variation in seed length (CV 10.43%). Only one population of *A. schoenoprasum* and *A. ubinicum* was studied, so no population-level comparison possible.

As a result of data processing, the external structures of four closely related species of the section *Schoenoprasum* were elucidated. The main morphological parameters of the seeds included the color, shape, surface texture, micropyle, chalaza, seed hilum, and suture (Table 3).

All seeds were small, triangular, and black with a rough, leathery surface. Mean seed size ranged from 2.95–3.50 mm in length, 1.25–1.85 mm in width, and 1.27–1.75 mm in thickness. *A. ivasczenkoae* and *A. ledebourianum* were moderately flattened with indented or pointed ends, *A. schoenoprasum* showed a flat base with pointed ends, and *A. ubinicum* displayed a strongly elongated, pointed apex and the most wrinkled surface. The micropyle was overgrown and barely visible in all species, while the chalaza was thickened and ribbed, with species-specific variation in prominence. The hilum was small, linear, and located either along the rib (*A. ivasczenkoae*, *A. ledebourianum*) or near the micropyle (*A. schoenoprasum*, *A. ubinicum*). The raphe was consistently short and faintly visible along the lateral edge.

### 3.2. Analysis of Morphology of Seed Traits

**Figure 4:**
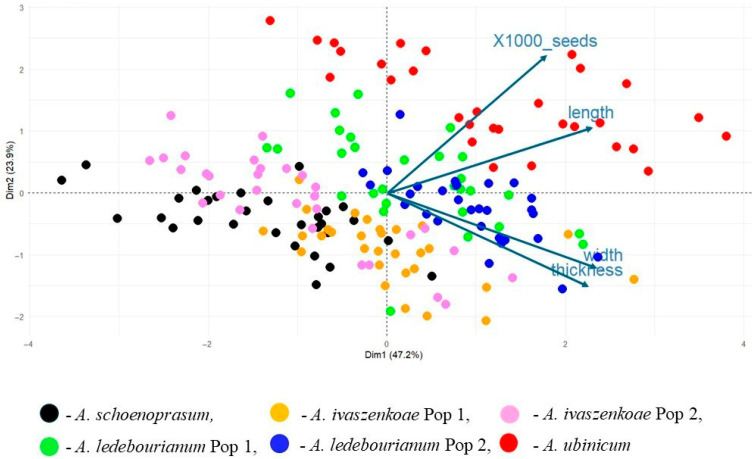
PCA based on seed morphological parameters of collected *Allium* seeds. Colored dots indicate six populations of four species.

The PCA based on the morphological parameters of individuals of the *Allium* species revealed a high level of diversity within the entire collection and also enabled the identification of clustering and differences among the collected species and populations (Figure 4).

Together, the two principal components explained 71.1% of the total variation (Dim1 = 47.2%, Dim2 = 23.9%). Three major clusters were clearly distinguished. The samples of *A. ubinicum* formed a distinct and well-separated cluster on the positive side of Dim1 and Dim2. Two populations of *A. ivaszenkoae* were positioned close to each other, forming a second cluster along with *A. schoenoprasum*. The samples of *A. ledebourianum* (green and blue) overlapped extensively, forming the third intermediate cluster between the first and the second ones. The PCA biplot also indicated strong positive correlations between seed width and thickness, while seed length was closely associated with 1000-seed weight. Among the studied taxa, *A. ubinicum* showed the highest values for seed length and 1000-seed weight, followed by *A. ledebourianum*, *A. ivaszenkoae*, and *A. schoenoprasum*.

**Figure 5:**
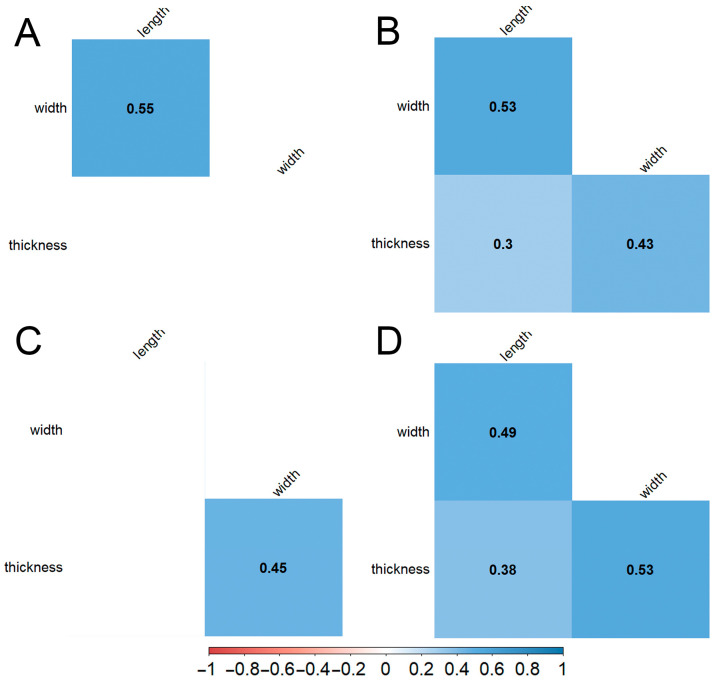
Correlation of quantitative parameters of seeds. (**A**)—*A. schoenoprasum*, (**B**)—*A. ivasczenkoae*, (**C**)—*A. ledebourianum*, (**D**)—*A. ubinicum*. Correlation coefficients with significance at *p* < 0.05 are highlighted. The color gradient represents the correlation strength: red indicates a strong negative correlation (1), blue indicates a strong positive correlation (1), and values near 0 (white) indicate little to no correlation.

A correlation analysis of the seed length, width, and thickness, performed separately for each species, showed a moderate positive correlation (r from 0.49 to 0.55 at *p* < 0.05) between the seed length and width for *A. schoenoprasum*, *A. ivasczenkoae*, and *A. ubinicum* (Figure 5A). A moderate positive correlation (r from 0.43 to 0.53 at *p* < 0.05) was also found between the seed width and thickness for *A. ivasczenkoae*, *A. ledebourianum*, and *A. ubinicum* (Figure 5A). A weak positive correlation (r from 0.30 to 0.38 at *p* < 0.05) was observed between the seed length and thickness for *A. ivasczenkoae* and *A. ubinicum* (Figure 5A).

### 3.3. Multivariate Analysis of Seed Traits

**Figure 6:**
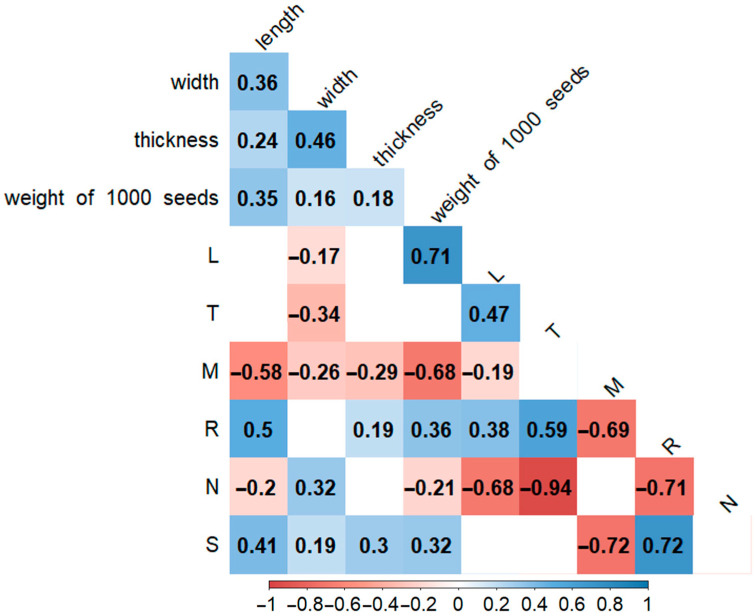
Correlation between quantitative parameters of seeds and environmental parameters. L—light, T—temperature, M—moisture, R—reaction, N—nutrients, S—salinity. Correlation coefficients with significance at *p* < 0.05 are highlighted. The color gradient represents the correlation strength: red indicates a strong negative correlation (1), blue indicates a strong positive correlation (1), and values near 0 (white) indicate little to no correlation.

Data from all four species were used for a correlation analysis of the seed size and weight in relation to environmental conditions (Table S1) (Figure 6).

Correlation analysis revealed several significant (*p* < 0.05) relationships between environmental parameters and quantitative seed traits (Figure 6). Negative associations were observed between light (L) and seed length, as well as between temperature (T) and all four traits (seed length, width, thickness, and 1000-seed weight). Nutrients (N) showed a negative correlation with seed length and 1000-seed weight. In contrast, positive correlations were found for moisture (M) with seed length, thickness, and 1000-seed weight, and for salinity (S). N also exhibited a positive correlation with seed width. Overall, these results indicate that temperature exerts a constraining effect on seed morphology, while moisture and salinity act as promoting factors, with nutrients showing a dual role depending on the seed parameter considered.

**Figure 7:**
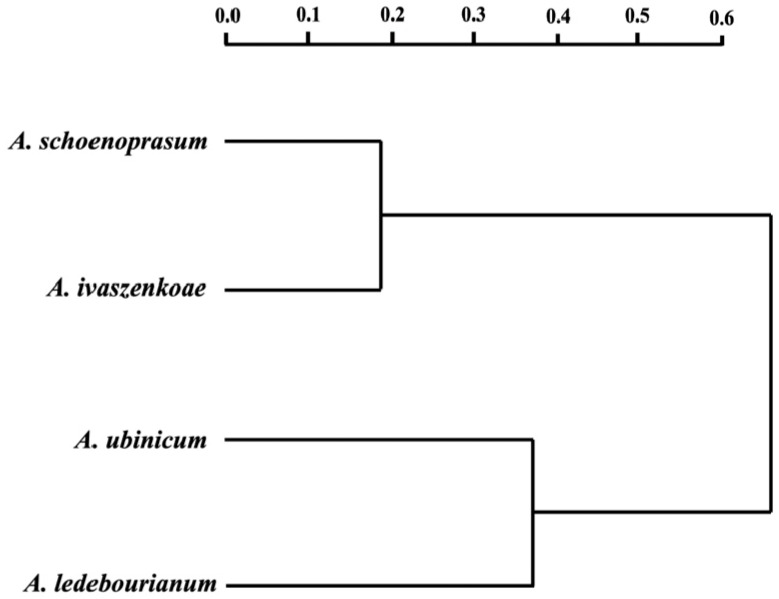
Dendrogram of interspecific similarities and differences of four *Allium* species of the *Schoenoprasum* section from Eastern Kazakhstan. The scale above the dendrogram represents the genetic distance.

To explore the interspecific similarities and differences based on the studied morphological parameters, a cluster dendrogram was constructed for species from the section *Schoenoprasum* (Figure 7).

The dendrogram indicated that the *A. schoenoprasum* seeds showed a high degree of similarity to those of *A. ivaszenkoae*, forming Cluster 1, while the *A. ubinicum* seeds appeared more similar to those of *A. ledebourianum*, forming Cluster 2 (Figure 7). This pattern is consistent with the PCA results (Figure 4), confirming the separation of the studied taxa into two major groups.

**Figure 8:**
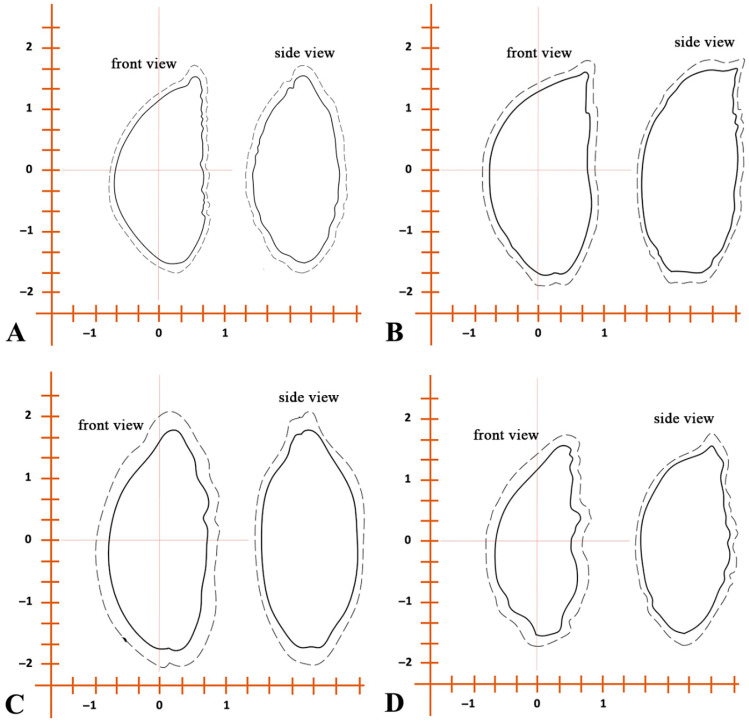
Standard external structure diagrams of species of section *Schoenoprasum* from Eastern Kazakhstan, based on average linear dimensions. Maximum dimensions are indicated by a dashed line. (**A**)—*A. ivasczenkoae*, (**B**)—*A. ledebourianum*, (**C**)—*A. ubinicum*, (**D**)—*A. schoenoprasum*.

The elliptic Fourier reconstructions of seed outlines (Figure 8) revealed clear interspecific variation in both front and side views. *A. schoenoprasum* and *A. ivaszenkoae* seeds were more rounded with less curvature, while *A. ledebourianum* and *A. ubinicum* displayed more elongated and asymmetrical profiles. These shape differences support the separation of species observed in the PCA and dendrogram analyses.

**Figure 9:**
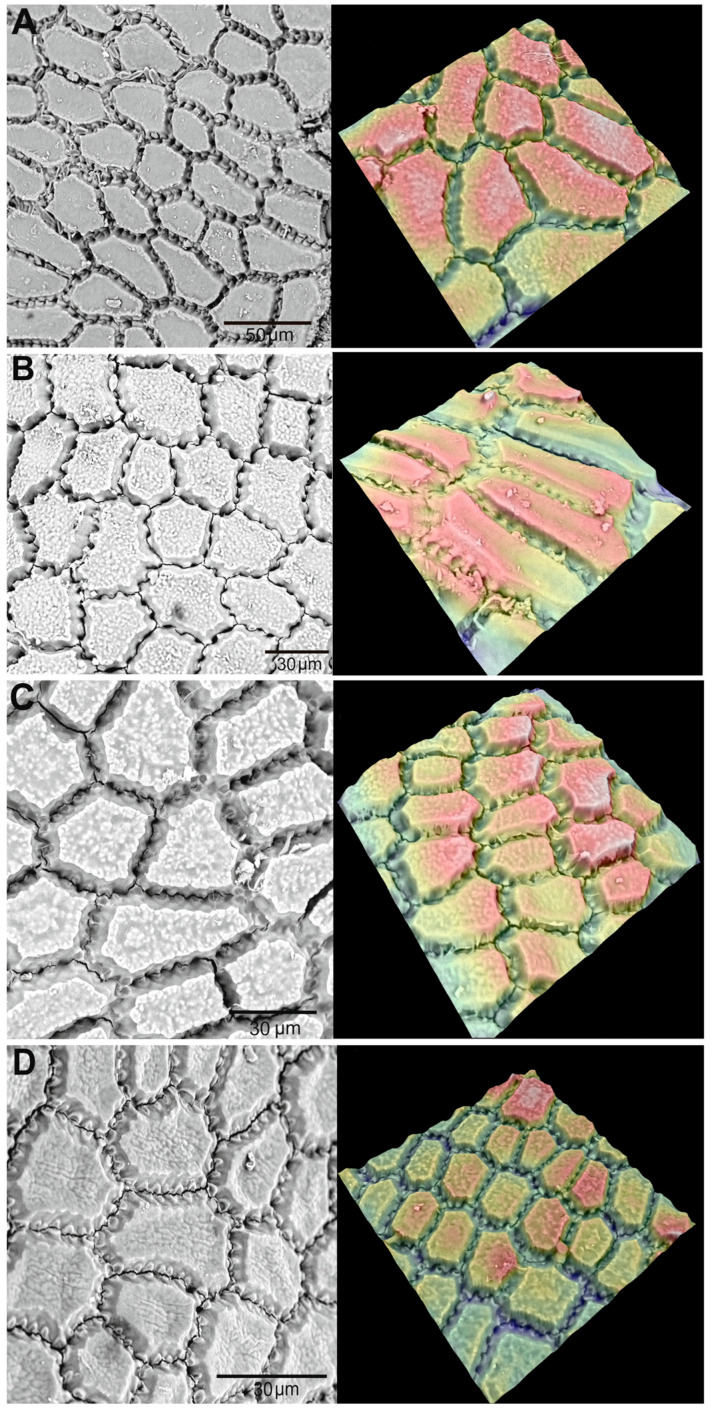
Seed coat micromorphology of four *Allium* species as seen under scanning electron microscope. (**A**)—*A. ledebourianum*, (**B**)—*A. ivasczenkoae*, (**C**)—*A. schoenoprasum*, (**D**)—*A. ubinicum*.

Analyzing the seed micromorphology of the studied species did not reveal any distinct differences that would allow for their unambiguous identification. The surface cells of the seeds exhibit contours with varying degrees of regularity ranging from slightly irregular to clearly polygonal, with a predominance of pentagonal and hexagonal forms (Figure 9). The anticlinal walls are distinctly undulated, whereas the periclinal walls are relatively smooth (Figure 9A,D) but covered with fine papillae (Figure 9B,C), giving the surface a slightly rough texture.

## 4. Discussion

One of the primary objectives of classical taxonomy is the identification and establishment of stable and qualitative diagnostic characteristics in the generative organs of plants. Among these, seed morphology holds particular taxonomic significance due to its relatively conservative nature and high diagnostic value. Seeds often retain morphological traits that are stable across environments, making them especially useful for species identification and differentiation, particularly in taxa where vegetative and floral traits may be variable or absent at certain stages of development [51,52].

The morphology of *Allium* seeds has been the focus of several detailed investigations. For example, Ulcay [20] examined the seed characteristics of two endemic Turkish species, *A. brevicaule* and *A. scorodoprasum* ssp. *rotundum*, while Celep et al. [22] conducted a broader survey encompassing 62 species across nine sections of *Allium* in Turkey. In Iran, Ebrahimi et al. [23] focused on the endangered *A. hirtifolium*, and in China, Lin and Tan [24] studied 38 species from 19 sections using seed surface structures. A more recent comprehensive study by Yusupov et al. [53] assessed phylogenetic relationships based on seed traits in 95 species from 58 sections. These comparative works provide important background but have largely treated seed morphology as a supplementary trait. By contrast, our results demonstrate the potential of seed characteristics as primary taxonomic markers, particularly within morphologically complex and closely related groups such as the section *Schoenoprasum*.

In our study, emphasis was placed not only on surface features [26,27,54] but also on micromorphological structures such as the hilum, chalaza, and micropyle. These seed-specific traits proved critical for distinguishing morphologically similar or rare species, especially in the absence of floral material. The morphometric assessment of seeds from four *Allium* species (*A. schoenoprasum*, *A. ledebourianum*, *A. ubinicum*, and *A. ivaszenkoae*) revealed notable interspecific variation. For instance, the significantly larger and heavier seeds of *A. ubinicum* compared to *A. schoenoprasum* may reflect ecological adaptations to specific habitats (Table 3). The PCA supported these observations, with *A. ubinicum* forming a well-separated cluster, while *A. ledebourianum* and *A. schoenoprasum* showed partial overlap (Figure 4). Similarly, elliptic Fourier descriptors quantified subtle shape differences: the rounded seeds of *A. schoenoprasum* and *A. ivaszenkoae* contrasted with the elongated and asymmetric profiles of *A. ledebourianum* and *A. ubinicum* (Figure 8). These patterns are consistent with both PCA and dendrogram analyses, strengthening the evidence for taxonomic distinctiveness within the section.

The correlations observed among morphometric traits provide additional insights. A positive correlation between seed width and thickness in *A. ubinicum* and *A. ivaszenkoae* may reflect shared evolutionary pressures or convergent adaptations to similar ecological conditions (Figure 5). Dendrogram clustering further supported these relationships, showing *A. schoenoprasum* and *A. ivaszenkoae* forming a tight cluster, while *A. ubinicum* grouped closer to *A. ledebourianum* (Figure 7). Such outcomes underscore the utility of seed morphology in resolving species boundaries, complementing traditional approaches that rely heavily on floral traits.

Importantly, comparisons between populations of the same species revealed that ecological differences can influence certain quantitative seed traits, although qualitative micromorphological characters remained stable. For instance, the two populations of *A. ivaszenkoae* displayed slight but consistent differences in seed length and 1000-seed weight (Table 3), which correlated with contrasting environmental parameters such as moisture and nutrient availability (Figure 6). Similarly, variation in *A. schoenoprasum* populations was reflected in seed width, aligning with local differences in salinity and soil reaction. Despite these quantitative shifts, traits such as testa cell wall position, hilum structure, and overall seed shape were conserved across populations, suggesting that these micromorphological features are less influenced by environment and thus more reliable as diagnostic markers. This dual pattern indicates that while quantitative traits may reflect adaptive ecological plasticity, qualitative attributes provide stable taxonomic signals, consistent with earlier studies on other *Allium* species [23,24].

Environmental influences should also be considered more broadly. The significant negative correlation between the length-to-thickness ratio (L/T) and seed length (Figure 6) suggests that external ecological pressures—such as soil type, elevation, or drought—may drive adaptive changes in seed compactness. Similar environment-driven seed modifications have been reported in other *Allium* species [23,24], indicating that seed traits can provide indirect insights into ecological strategies and reproductive success.

An important implication of our findings concerns the taxonomic status of *A. ubinicum*. Previous authors suggested synonymizing it with *A. schoenoprasum*. However, the distinct morphometric and clustering patterns observed here argue against such synonymization. Instead, our results support its recognition as a distinct species, though we acknowledge that additional morphological, ecological, and molecular data will be necessary to confirm this status as it was proposed by Kotkuhov et al. [46].

The diagnostic value of the analyzed seed attributes varied among the studied taxa. Among the quantitative traits, seed length and 1000-seed weight proved to be the most informative for distinguishing between species, as they showed consistent differentiation, particularly in *A. ubinicum*, which displayed the largest seeds and highest weight values. Seed thickness and width, while correlated, provided additional support in discriminating closely related taxa such as *A. schoenoprasum* and *A. ivaszenkoae*. Qualitative attributes (shape, testa surface patterns, and color) further enhanced species delimitation, confirming their established value as taxonomically informative traits in *Allium* [22].

From a broader perspective, the integration of seed morphology into taxonomic frameworks offers significant implications for both systematics and conservation. For rare or endemic species, where flowering stages are often sporadic or absent, seed traits provide reliable diagnostic markers [26,53,55]. This approach could also aid in the development of more precise identification keys for section *Schoenoprasum* and other sections within the genus *Allium*. Moreover, such traits may serve as a foundation for phylogenetic inferences, as demonstrated in other groups [53].

Despite these contributions, the present study has certain limitations. The sample size was restricted to four species, which, although representative, does not encompass the full diversity of section *Schoenoprasum*. Environmental influences on seed morphology were inferred but not directly tested, and molecular phylogenetic data were not integrated into the current framework. Future research should expand sampling across more taxa, combine seed morphological traits with genomic data, and evaluate environmental effects experimentally to disentangle plasticity from genetic control.

In summary, our study demonstrates that seed morphology represents a robust and underutilized taxonomic tool in *Allium*. Larger and heavier seeds of *A. ubinicum* reflect regional divergence or adaptation, supporting its recognition as a distinct taxon. By combining morphometric analyses, correlation studies, PCA, and clustering approaches, we provide a comprehensive view of species relationships and highlight the importance of seed traits for taxonomy, ecology, and conservation. These findings call for broader integration of seed-based traits into systematic and evolutionary studies, ultimately contributing to more accurate classification and better-informed conservation strategies.

## 5. Conclusions

This study provides the first comprehensive evidence that seed morphology and quantitative morphometrics hold substantial taxonomic value for species delimitation within the section *Schoenoprasum* of *Allium*. Our analyses (PCA, correlation assessment, and hierarchical clustering) revealed marked interspecific differences in seed size, shape, and weight, with *A. ubinicum* consistently forming a distinct morphological cluster. This finding highlights the novelty of our work, as it supports the recognition of *A. ubinicum* as a separate taxon and challenges its long-standing synonymization with *A. schoenoprasum*. The observed correlation patterns among seed traits suggest interactions between genetic and environmental factors, indicating possible adaptive differentiation across ecological gradients. Although seed coat micromorphology alone proved insufficient for unequivocal delimitation, features such as the hilum, chalaza, and micropyle emerged as reliable generative traits for distinguishing closely related taxa. Beyond their taxonomic relevance, these results carry important conservation implications: accurate species delimitation is essential for assessing the status of localized and potentially vulnerable taxa, such as *A. ubinicum*, and for prioritizing their protection in the ecologically sensitive Kazakhstan Altai. Taken together, our study underscores the novelty and diagnostic power of seed traits in *Allium* taxonomy and advocates for their broader integration with molecular and environmental data to advance both systematics and conservation of this genus.

## Figures and Tables

**Table 1:** Description of population locations of 4 *Allium* species of the section *Schoenoprasum*, found in Eastern Kazakhstan.

Population	Coordinates	Collection Site and Habitat Description	Area, m^2^
*A. ledebourianum* Schult. et Schult.			
Pop 1	50.3422410 83.7344190	Kazakhstan, East Kazakhstan region, Ivanovsky ridge, foothills of Listvyazhnaya mountain, hummocky bogs	300
Pop 2	50.358797 83.901374	Kazakhstan, East Kazakhstan region, Ivanovsky ridge, Bolshaya Poperechka river valley, Gray Lug tract, grassy meadow	150
*A*. *ivasczenkoae* Kotuch.			
Pop 1	50.287624 83.304463	Kazakhstan, East Kazakhstan region, Ivanovsky ridge, Kozlushka mountain, swampy clearing in willow	50
Pop 2	50.379005 83.791850	Kazakhstan, East Kazakhstan region, Ivanovsky ridge, foothills of Listvyazhnaya mountain, hummocky bogs	250
*A. ubinicum* Kotuch.			
Pop 1	50.567472 82.109523	Kazakhstan, East Kazakhstan region, Ubinsky ridge, Uba River valley, along wet banks	200
*A*. *schoenoprasum* L.			
Pop 1	49.443428 82.723457	Kazakhstan, East Kazakhstan region, Kalbinsky ridge, Sibinskaya depression, flood meadows	1000

**Table 2:** Morphometric characteristics of seeds of *Allium* species within the section *Schoenoprasum* from Eastern Kazakhstan.

Population	Length, mm			Width, mm			Thickness, mm			Weight of 1000 Seeds, g
	M ± SE Min–Max	Cv, %	P%	M ± SE Min–Max	Cv, %	P%	M ± SE Min–Max	Cv, %	P%	
*Allium ledebourianum*										
Pop 1	3.036 ± 0.093 2.25–3.50	7.932	1.448	1.539 ± 0.055 1.30–1.80	9.345	1.706	1.508 ± 0.037 1.30–1.70	6.442	1.175	1.201
Pop 2	3.268 ± 0.055 3.10–3.57	4.448	0.812	1.605 ± 0.041 1.35–1.82	6.651	1.214	1.558 ± 0.035 1.35–1.70	5.898	1.070	1.116
*Allium ivasczenkoae*										
Pop 1	3.271 ± 0.081 2.95–3.90	6.499	1.187	1.537 ± 0.049 1.25–1.90	8.464	1.545	1.534 ± 0.036 1.35–1.75	6.109	1.115	0.602
Pop 2	3.059 ± 0.063 2.75–3.35	5.377	0.981	1.466 ± 0.048 1.25–1.65	8.515	1.555	1.386 ± 0.059 1.20–1.70	11.098	2.026	0.955
*Allium ubinicum*										
Pop 1	3.438 ± 0.137 3.05–4.00	10.427	1.904	1.487 ± 0.062 1.17–1.85	10.870	1.985	1.505 ± 0.049 1.27–1.70	8.544	1.559	1.350
*Allium schoenoprasum*										
Pop 1	3.025 ± 0.084 2.60–3.40	7.299	1.333	1.382 ± 0.051 1.15–1.65	9.701	1.771	1.434 ± 0.038 1.20–1.62	6.999	1.278	0.883

M—mean value, SE—standard error, min-max—minimum and maximum values, Cv—coefficient of variation, P%—the relative error of the sample mean.

**Table 3:** Morphological characteristics of seeds of *Allium* species within the section *Schoenoprasum* from Eastern Kazakhstan.

Morphological Traits	Species			
	*A. ivasczenkoae*	*A. ledebourianum*	*A. schoenoprasum*	*A. ubinicum*
Size, shape	Small (parvum), triangular, straight	Small (parvum), triangular, straight	Small (parvum), triangular, straight	Small (parvum), triangular, straight
Length, mm	2.95–3.90 (mean 3.27)	2.25–3.50 (mean 3.04)	2.60–3.40 (mean 3.025)	3.05–4.00 (mean 3.438)
Width, mm	1.25–1.90 (mean 1.54)	1.30–1.80 (mean 1.53)	1.15–1.65 (mean 1.382)	1.17–1.85 (mean 1.487)
Thickness, mm	1.35–1.75 (mean 1.53)	1.30–1.70 (mean 1.51)	1.20–1.62 (mean 1.434)	1.27–1.70 (mean 1.505)
Weight of 1000 seeds, g	0.602	1.201	0.883	1.350
Seed color	Black, solid, matte	Black, solid, matte	Black, solid, matte	Black, solid, matte
Seed shape	Noticeably flattened. Triangular, with clearly defined curved edges. The base is significantly concave inward into the seed. One end of the seed is usually rounded; the other end is pointed.	Triangular, elongated. One end of the seed is more rounded and widened; the opposite end is sharply pointed and narrowed. The back is convex, the base is flat, slightly concave inward into the seed.	Triangular, has a flat base, slightly indented into the seed. The lateral edges are also slightly indented into the endosperm. The edges are curved and sinuous. Both ends of the seed are pointed.	Triangular, has a strongly elongated pointed end. The other end of the seed is slightly rounded. The lateral edges are pressed inward. The edges are slightly curved and pointed.
Seed surface	Rough, dry, leathery, slightly wrinkled, fine-grained.	Rough, dry, leathery, slightly wrinkled on the back and smooth at the base.	Rough, dry, leathery, wrinkled on the back, and smoother at the base.	Rough, dry, leathery, heavily wrinkled.
Micropyle	Overgrown, barely visible, triangular in shape, located at the narrow end of the seed.	Overgrown, distinct, triangular in shape, located at the narrow end of the seed.	Overgrown, barely visible, dot-shaped, located at the narrow end of the seed.	Overgrown, poorly distinguishable, dot-shaped, located at the narrow end of the seed.
Chalaza	Noticeably thickened, strongly ribbed, located in the basal part.	Thickened, convex, ribbed, located in the basal part.	Slightly thickened, significantly ribbed, located in the basal part.	Slightly thickened, ribbed, located in the basal part.
Seed hilum	Small, linear, protruding, longitudinally slit-shaped, located along the rib in the middle part.	Small, linear, longitudinally slit-shaped, slightly protruding, close to the micropyle, located on the edge.	Small, linear, oval-shaped, slightly protruding, close to the micropyle, located on the edge.	Small, linear, longitudinally slit-shaped, slightly protruding, close to the micropyle, located on the edge.
Raphe (seed suture)	Barely noticeable, smooth, shortened, located on the lateral edge.	Faintly visible, slightly rough, located on the lateral edge.	Hardly visible, shortened, located on the lateral edge.	Hardly visible, shortened, located on the lateral edge.

## Data Availability

All data generated and analyzed during this study are included in the main text and Supplementary Materials.

## Supplementary Materials

The following supporting information can be downloaded at: https://www.mdpi.com/article/10.3390/biology14091230/s1, Table S1: Ellenberg Indicator Values for *A. ledebourianum*, *A. ivasczenkoae*, *A. schoenoprasum*, and *A. ubinicum*.

## Abbreviations

The following abbreviations are used in this manuscript:

SEM	Scanning electron microscopy
EIVs	Ellenberg Indicator Values
