# Supplementary material for: Seed Morphology of Allium L. Endemic Species from Section Schoenoprasum (Amaryllidaceae) in Eastern Kazakhstan
Source: Biology (Basel). 2025 Sep 9;14(9):1230. doi: 10.3390/biology14091230 (PMC12467962; doi:10.3390/biology14091230)
Supplement: Supplementary file 1 [file biology-14-01230-s001.zip › biology-3827632-supplementary.pdf]

Species, population	L	T	M	R	N	S
Allium ledebourianum Schult. et Schult.						
Pop 1	8	4.5	8.5	4.5	7.5	2.5
Pop 2	7.5	4	7.5	5	8	4
A. ivasczenkoae Kotuch.						
Pop 1	7	5	8	5	7	3
Pop 2	7.5	4.5	8.5	4.5	7.5	2.5
A. ubinicum Kotuch.						
	8.5	5.5	7	5.5	4.5	3.5
A. schoenoprasum L.						
	8	5.5	9	5	5	3

Verbal definitions of the scales of Ellenberg indicator values.

L – Light (scale 1–9)

1 – deep shade plant, occurring where the incident diffuse radiation is less than 1% of that in an open area, rarely at more than 30%
2 – between 1 and 3
3 – shade plant, usually occurring where the incident diffuse radiation is less than 5% of that in an open area, but also at sunnier sites
4 – between 3 and 5
5 – semi-shade plant, only exceptionally occurring in full light, but usually at more than 10% of the diffuse radiation incident in an open area
6 – between 5 and 7; rarely at less than 20% of diffuse radiation incident in an open area
7 – half-light plant, mostly occurring at full light, but also in the shade up to about 30% of diffuse radiation incident in an open area
8 – light plant, only exceptionally occurring at less than 40% of diffuse radiation incident in an open area
9 – full light plant, occurring only in fully irradiated places, not at less than 50% of diffuse radiation incident in an open area

T – Temperature (scale 1–9)

1 – cold indicator, only in high mountain areas, i.e. the alpine and nival belts
2 – between 1 and 3 (many alpine species)
3 – cool indicator, mainly in subalpine areas
4 – between 3 and 5 (especially high montane and montane species)
5 – moderate heat indicator, from lowland to montane belt, mainly in submontane-temperate areas
6 – between 5 and 7 (lowland and colline species)
7 – heat indicator, occurring in relatively warm lowlands
8 – between 7 and 9
9 – extreme heat indicator

M – Moisture (scale 1–12)

1 – strong drought indicator, viable at sites that frequently dry out and confined to dry soils
2 – between 1 and 3
3 – missing on damp soil
4 – between 3 and 5
5 – indicator of fresh soils, focus on soils of average moisture, missing on wet soils and on soils that frequently dry out
6 – between 5 and 7
7 – humidity indicator, focus on well moistened, but not wet soils
8 – between 7 and 9
9 – wetness indicator, focus on often soaked, poorly aerated soils
10 – aquatic plant that survives long periods without soil flooding
11 – aquatic plant rooted under water, but at least temporarily with leaves above the surface, or a plant floating on the water surface
12 – permanently or almost permanently submerged aquatic plant

R – pH reaction (scale 1–9)

1 – indicator of strong acidity, never occurring in slightly acidic to alkaline conditions

2 – between 1 and 3

3 – acidity indicator, occurring mainly in acidic conditions, exceptionally in neutral conditions

4 – between 3 and 5

5 – indicator of moderate acidity, occurring rarely in strongly acidic as well as in neutral to alkaline conditions

6 – between 5 and 7

7 – indicator of slightly acidic to slightly basic conditions, never occurring in very acidic conditions

8 – between 7 and 9, occurring mostly in calcium-rich conditions

9 – base and lime indicator, always occurring in calcium-rich conditions

N – Nutrients (scale 1–9)

1 – occurring at nutrient-poorest sites

2 – between 1 and 3

3 – occurring at nutrient-poor sites more frequently than at average sites and exceptionally at rich sites

4 – between 3 and 5

5 – occurring at moderately nutrient-rich sites, and less frequently at poor and rich sites

6 – between 5 and 7

7 – occurring at nutrient-rich sites more often than at average sites and only exceptionally at poor sites

8 – pronounced nutrient indicator

9 – concentrated at very nutrient-rich sites

S – Salinity (scale 0–9)

0 – not salt tolerant, glycophyte

1 – salt tolerant, mostly on low-salt to salt-free soils, but occasionally on slightly salty soils

2 – oligohaline, often on soils with very low salt content

3 – -mesohaline, mostly on soils with low salt content

4 – /-mesohaline, mostly on soils with low to moderate salt content

5 – -mesohaline, mostly on soils with a moderate salt content

6 – -meso/polyhaline, on soils with moderate to high salt content

7 – polyhaline, on soils with a high salt content

8 – euhaline, on soils with a very high salt content

9 – euhaline to hypersaline, on soils with a very high and in dry periods extremely high salt content
